# Editorial: Quo Vadis Lipid Mediators – Lipid Mediators Implication in Inflammation and Chronic Inflammatory Diseases

**DOI:** 10.3389/fimmu.2021.699276

**Published:** 2021-05-12

**Authors:** Danuta Radzioch, Martin Giera, Juan Bautista De Sanctis

**Affiliations:** ^1^ Department of Experimental Medicine and Department of Genetics, The Research Institute of the McGill University Health Centre, McGill University, Montreal, QC, Canada; ^2^ Metabolomics Group, Leiden University Medical Center, Leiden, Netherlands; ^3^ Institute of Molecular and Translational Medicine, Faculty of Medicine and Dentistry, Palacky University, Olomouc, Czechia

**Keywords:** neutrophils, nuclear receptor corepressor 1, orsomucoid like 3, asthma, Alzheimer’s disease, w 3 fatty acids, sebocytes, inflammation

Lipids are involved in the induction, resolution, and chronicity of immune responses. In this thematic issue, original and review articles have been devoted to studying different inflammation and immune response areas. Li et al. analyzed the RNA present in exosomes from the plasma chronic coronary artery disease (CAD) patients and its role in different macrophage populations related to atherosclerosis. An association among transcriptional signature and macrophage subpopulations in the atherosclerosis microenvironment was documented.

Souza et al. studied the importance of GPR40, a free fatty acid G protein-coupled receptor, by using the agonist GW9508. The authors were able to show that the expression and function of the receptor were enhanced upon activation of neutrophils. Upon activation, neutrophils phagocyte bacteria more efficiently and produce pro-resolving lipid mediators to decrease the inflammatory burden.

The effect of docosahexaenoic acid (DHA) on the interaction between NK cells and neutrophil was analyzed by Jensen et al. DHA decreased NK induced neutrophil activation, and vice versa, NK activation induced by neutrophils. These results suggest that DHA has an anti-inflammatory role in the NK neutrophil interaction, critical in several pathophysiological processes.

Geiger et al. analyzed the Nuclear Receptor Corepressor 1 (NCOR1) association with immune metabolic diseases and atherosclerosis. NCOR1 is involved in the modulation of lipid catabolism and anabolism and the expression of proinflammatory mediators. Regulation of this repressor is essential in several cellular pathways involved in chronicity and inflammatory response.

In critically ill patients, lipidomics can provide essential information regarding a patient’s ability to overcome an acute inflammatory response (Cioccari et al.). The role of pro-resolving lipid mediators’ might be critical, and lipidomics could be used as an essential clinical parameter in the ICU, as concluded by Cioccari et al.

Two reviews focused on asthma and lipid metabolism. The first review deals with the effect of arachidonic acid-derived pro-resolving mediators and their therapeutic applicability (Insuela et al.). Even though several of these metabolites suppress the immune response in asthma in animal models, the low stability and side effects of these metabolites have hampered possible therapeutic application. Novel compounds with improved pharmacochemical properties must meet the stability requirements necessary for their use approval by regulatory agencies.

Orsomucoid like 3 (ORMDL3) is an endoplasmic reticulum transmembrane protein that controls sphingolipid biosynthesis by regulating the enzyme serine palmitoyltransferase (SPT). Luthers et al. reviewed the importance of ORMDL3 in T cell responses in asthma.

The role of sphingolipids is also reviewed in Alzheimer’s disease by De Wit et al. The role of proinflammatory and pro-resolving lipid mediators is examined in this complex and incurable disease.

Dasilva et al. studied the effect of fish oil diet on white adipose tissue of prediabetic rats. They show that an increase in polyunsaturated w3 fatty acid in adipose tissue parallels the production of pro-resolving mediators, decreasing the inflammatory burden, critical in this subclinical disease.

Finally, the contribution of Törőcsik in their original research analyzed how the role of palmitic acid-induced lipid accumulation and inflammation in the sebocytes can be modulated by epidermal growth factor. This research highlights the importance of sebaceous immunology, an interesting topic nowadays.

As editors of this Research Topic, we hope that the articles mentioned above will motivate researchers and clinicians to study lipid metabolism in the inflammatory response. We hope that lipidomics analysis will find its way into clinical routines as an integral part of personalized medicine.

## Author Contributions

All authors contributed equally. All authors contributed to the article and approved the submitted version.

## Funding

DR and JS are researchers funded by the grant from the Ministry of Education, Youth and Sport, Czech Republic: Molecular and Cellular Clinical Approach to Healthy Ageing, ENOCH (European Regional Development Fund Project No. CZ.02.1.01/0.0/0.0/16_019/0000868, IMTM #869/V19). DR is also funded by Prostate Canada grant TAG2017 Movember Translation Acceleration grant entitled: Translational procedure and interventional platform development for optimal delivery of therapeutics to prostate cancer based on magnetotactic bacteria; and grants from the Ministère de L’ Économie et de l’ Innovation: The enhancement of Cystic Fibrosis Transmembrane Conductance Regulator (CFTR) functional expression by Fenretinide (MESI Accounts 2768 and 3335).

## Conflict of Interest

The authors declare that the research was conducted in the absence of any commercial or financial relationships that could be construed as a potential conflict of interest.

